# Treatment of Obesity with Thyroid hormones in Europe. Data from the THESIS* Collaboration

**DOI:** 10.1007/s40618-024-02409-z

**Published:** 2024-06-15

**Authors:** J. C. Galofré, J. J. Díez, R. Attanasio, E. V. Nagy, R. Negro, E. Papini, P. Perros, M. Žarković, E. Akarsu, M. Alevizaki, G. Ayvaz, T. Bednarczuk, B. N. Beleslin, E. Berta, M. Bodor, A. M. Borissova, M. Boyanov, C. Buffet, M. C. Burlacu, H. Dobnig, V. Fadeyev, B. C. T. Field, E. Fliers, D. Führer, T. Hakala, J. Jiskra, P. Kopp, M. Krebs, M. Kršek, M. Kužma, M. Lantz, I. Lazúrová, L. Leenhardt, V. Luchytskiy, F. M. Puga, A. McGowan, S. Metso, C. Moran, T. Morgunova, D. A. Niculescu, B. Perić, T. Planck, C. Poiana, E. Robenshtok, P. O. Rosselet, M. Ruchala, K. R. Riis, A. Shepelkevich, M. Tronko, D. Unuane, I. Vardarli, W. E. Visser, M. Vryonidou, Y. R. Younes, L. Hegedüs

**Affiliations:** 1 Department of Endocrinology and Nutrition, Clínica Universidad de Navarra, Pío XII, 36., 31080 Pamplona, Spain; 2 Instituto de Investigación Sanitaria de Navarra (IdiSNA), Pamplona, Spain; 3 Thyroid Task Force From the Sociedad Española de Endocrinología y Nutrición (SEEN), Madrid, Spain; 4 Department of Endocrinology, Hospital Universitario Puerta de Hierro Majadahonda, Madrid, Spain; 5 Instituto de Investigación Sanitaria Puerta de Hierro Segovia de Arana, Majadahonda, Madrid Spain; 6 Department of Medicine, Universidad Autónoma de Madrid, Madrid, Spain; 7 Scientific Committee Associazione Medici Endocrinologi, Milan, Italy; 8 Division of Endocrinology, Department of Medicine, Faculty of Medicine, University of Debrecen, Debrecen, Hungary; 9 Division of Endocrinology, V. Fazzi Hospital, Lecce, Italy; 10 Department of Endocrinology and Metabolism, Regina Apostolorum Hospital, Rome, Italy; 11 Institute of Translational and Clinical Research, Newcastle University, Newcastle Upon Tyne, UK; 12 Faculty of Medicine, University of Belgrade, Belgrade, Serbia; 13 Division of Endocrinology, Department of Internal Medicine, Faculty of Medicine, Gaziantep University, Gaziantep, Turkey; 14 Endocrine Unit and Diabetes Centre, Department of Clinical Therapeutics, School of Medicine, Alexandra Hospital, National and Kapodistrian University of Athens, Athens, Greece; 15 Department of Endocrinology and Metabolism, Koru Ankara Hospital, Kizilirmak, Ankara, Turkey; 16 Department of Internal Medicine and Endocrinology, Medical University of Warsaw, Warsaw, Poland; 17 Institute of Endocrinology, Diabetes and Metabolic Disorders, Univeristy Clinical Center of Serbia, Belgrade, Serbia; 18 Clinic of Endocrinology and Metabolism, Medical Faculty, University Hospital “Sofiamed”, Sofia University “Saint Kliment Ohridski”, Sofia, Bulgaria; 19 Clinic of Endocrinology and Metabolism, University Hospital “Alexandrovska”, Sofia, Bulgaria; 20 Department of Internal Medicine, Medical University Sofia, Sofia, Bulgaria; 21 Thyroid Disease and Endocrine Tumor Department, Sorbonne Universitè, Hôpital Pitié-Salpêtriére, Paris, France; 22 Department of Endocrinology Diabetology and Nutrition, Cliniques Universitaires St-Luc, Université Catholique de Louvain, Brussels, Belgium; 23 Thyroid and Osteoporosis Praxis, Kumberg, Austria; 24 Thyroid Practice for Radiofrequency Ablation, Vienna, Austria; 25 Department of Endocrinology No. 1, N.V. Sklifosovsky Institute of Clinical Medicine, I.M. Sechenov First Moscow State Medical University, Moscow, Russian Federation; 26 Section of Clinical Medicine, Faculty of Health & Medical Sciences, University of Surrey, Guildford, Surrey UK; 27 Department of Endocrinology & Metabolism, Amsterdam UMC, University of Amsterdam, Amsterdam, The Netherlands; 28 Department of Endocrinology, Diabetes and Metabolism, University Hospital Essen, University-Duisburg-Essen, Essen, Germany; 29 Department of Surgery, Tampere University Hospital, Tampere, Finland; 30 3rd Department of Medicine, 1st Faculty of Medicine, Charles University, General University Hospital, Prague, Czech Republic; 31 Division of Endocrinology, Diabetes and Metabolism, University of Lausanne, Lausanne, Switzerland; 32 Internal Medicine III, Division of Endocrinology and Metabolism, Medical University of Vienna, Vienna, Austria; 33 5th Department of Internal Medicine, Medical Faculty of Comenius, University and University Hospital, Bratislava, Slovakia; 34 Department of Endocrinology, Skåne University Hospital, Malmö, Sweden; 35 1st Department of Internal Medicine of the Medical Faculty, P.J. Šafárik University Košice, Košice, Slovakia; 36 Department of Reproductive Endocrinology, Institute of Endocrinology and Metabolism Named after V.P. Komissarenko, National Academy of Medical Science of Ukraine, Kyiv, Ukraine; 37 Endocrinology, Diabetes and Metabolism Service, Centro Hospitalar Universitário de Santo António, Porto, Portugal; 38 Robert Graves Institute, Tallaght University Hospital, Dublin, Ireland; 39 Department of Endocrinology, Tampere University Hospital, Tampere, Finland; 40 Diabetes & Endocrinology Section, Beacon Hospital, Beacon Court, Dublin, Ireland; 41 School of Medicine, University College Dublin, Dublin, Ireland; 42 Department of Endocrinology, Carol Davila University of Medicine and Pharmacy, Bucharest, Romania; 43 Department of Endocrinology, Diabetes and Metabolic Diseases “Mladen Sekso”, University Hospital Center “Sisters of Mercy”, Zagreb, Croatia; 44 Thyroid Cancer Service, Endocrinology and Metabolism Institute, Beilinson Hospital and Davidoff Cancer Center, Rabin Medical Center, Sackler Faculty of Medicine, Tel Aviv University, Petah Tikva, Israel; 45 Cabinet Médical 2, Rue Bellefontaine, Lausanne, Switzerland; 46 Department of Endocrinology, Metabolism and Internal Medicine, Poznan University of Medical Sciences, Poznań, Poland; 47 Department of Endocrinology, Odense University Hospital, Odense, Denmark; 48 Department of Endocrinology, Belarusian State Medical University, Minsk, Republic of Belarus; 49 V.P. Komisarenko Institute of Endocrinology and Metabolism of Academy of Medical Sciences of Ukraine, Kyiv, Ukraine; 50 Department of Internal Medicine, Endocrine Unit, UZ Brussel, Vrije Universiteit Brussel, Brussels, Belgium; 51 Department of Medicine I, Klinikum Vest GmbH, Knappschaftskrankenhaus Recklinghausen, Academic Teaching Hospital, Ruhr-University Bochum, Recklinghausen, Germany; 52 5th Medical Department, Division of Endocrinology and Diabetes, Medical Faculty Mannheim, Heidelberg University, Mannheim, Germany; 53 Rotterdam Thyroid Center, Department of Internal Medicine, Erasmus Medical Center, Rotterdam, The Netherlands; 54 Department of Endocrinology and Diabetes Centre, Hellenic Red Cross Hospital, Athens, Greece; 55 East Surrey Hospital, Surrey & Sussex Healthcare NHS Trust, Redhill, Surrey UK

**Keywords:** Obesity, Hypothyroidism, Levothyroxine, Survey

## Abstract

**Purpose:**

The use of thyroid hormones (TH) to treat obesity is unsupported by evidence as reflected in international guidelines. We explored views about this practice, and associations with respondent characteristics among European thyroid specialists.

**Methods:**

Specialists from 28 countries were invited to a survey via professional organisations. The relevant question was whether “*Thyroid hormones may be indicated in biochemically euthyroid patients with obesity resistant to lifestyle interventions”*.

**Results:**

Of 17,232 invitations 5695 responses were received (33% valid response rate; 65% women; 90% endocrinologists). Of these, 290 (5.1%) stated that TH may be indicated as treatment for obesity in euthyroid patients. This view was commoner among non-endocrinologists (8.7% vs. 4.7%, p < 0.01), private practice (6.5% vs. 4.5%, p < 0.01), and varied geographically (Eastern Europe, 7.3%; Southern Europe, 4.8%; Western Europe, 2.7%; and Northern Europe, 2.5%). Respondents from Northern and Western Europe were less likely to use TH than those from Eastern Europe (p < 0.01). Gross national income (GNI) correlated inversely with this view (OR 0.97, CI: 0.96–0.97; p < 0.001). Having national guidelines on hypothyroidism correlated negatively with treating obesity with TH (OR 0.71, CI: 0.55–0.91).

**Conclusions:**

Despite the lack of evidence, and contrary to guidelines’ recommendations, about 5% of respondents stated that TH may be indicated as a treatment for obesity in euthyroid patients resistant to life-style interventions. This opinion was associated with (i) respondent characteristics: being non-endocrinologist, working in private practice, treating a small number of hypothyroid patients annually and (ii) national characteristics: prevalence of obesity, Eastern Europe, low GNI and lack of national hypothyroidism guidelines.

**Supplementary Information:**

The online version contains supplementary material available at 10.1007/s40618-024-02409-z.

## Introduction

Treatment of Hypothyroidism in Europe by Specialists: An International Survey (THESIS) is a large-scale European study aiming to explore views about the use of thyroid hormones (TH) among thyroid specialists in Europe. It was completed in 2021, and twenty countries have already reported their national data [1–20].

THESIS has allowed the evaluation of real-life practices by thyroid specialists that include both evidence-based indications for TH use and that of nonconventional use [21, 22]. Here we report the aggregate data with respect to the use of TH in obese euthyroid individuals resistant to lifestyle interventions.

The rationale behind using of TH in euthyroid individuals with obesity was based in a few relevant experimental and clinical observations. It is well recognised that TH have important thermogenic effects that promote caloric expenditure [23]. Hypothyroid patients are prone to weight gain, mostly due to fluid retention, which may decrease following treatment for hypothyroidism [24]. In addition, body mass index (BMI) and body weight are typically positively associated with serum thyrotropin (TSH) levels [24, 25], although this relationship seems to be somewhat modulated by autoimmunity [26, 27]. Nevertheless, no study has demonstrated that TH are effective for weight loss in euthyroid individuals with obesity without causing side effects [24, 28]. On the contrary, TH induces iatrogenic thyrotoxicosis [24, 28] that is associated with increased somatic as well as psychiatric morbidity and excess mortality [29–32], and does not improve quality of life [33].

We aimed to document the respondent characteristics and views of thyroid specialists members of 28 national European Endocrine Societies about the use of TH in euthyroid subjects with obesity resistant to lifestyle intervention.

## Material and methods

Guidelines for internet-based electronic surveys (CHERRIES) were followed. The survey recruited thyroid specialists who were members of national endocrine and/or thyroid scientific professional organizations from European countries with more than 4 million citizens. The project was supervised by a Steering Committee (LH, EVN, EP, PP, RA, and RN).

The survey was conducted between 2019 and 2021 and details are reported elsewhere [21, 22]. In brief, the anonymous online questionnaire comprised of eight questions about physician characteristics and twenty-three questions about the use of TH in various clinical scenarios. The survey link was distributed through national thyroid or endocrine professional societies. Two national leads of each country and the Steering Committee were responsible for the authenticity of the data received. The relevant question for obesity was whether “*Thyroid hormones may be indicated in biochemically euthyroid patients with obesity resistant to lifestyle interventions”.* The questionnaire was developed in English and the survey was translated into the national language at the discretion of the national leads.

After completing the survey, national leads reported if their country had at the time of the survey (i) national guidelines for thyroid disease, including management of hypothyroidism, and (ii) guidelines for the management of obesity. In the absence of national guidelines, national leads were asked whether any specific international guideline was officially recommended by the national society and whether such guidelines support or discourage the use of TH for obesity.

### Statistical analyses

Responses that contained complete information about the respondent’s demographics were considered as valid and included in the analysis.

Statistical calculations were performed with R [34]. Due to the characteristics of the information, the survey data were not weighted. The information referring to qualitative and quantitative variables is presented in frequencies or proportions, and means with standard deviations, respectively. The association between qualitative variables was calculated using chi-square and Cramer’s tests. Linear, logistic and ordinal regression was performed when applicable [35], with the statistical and ordinal R packages. The statistical significance level was set at 5%. The effect size is independent of the sample size and the p-value, allowing us to rule out statistically significant but practically irrelevant results [36, 37]. Therefore, we reported both p-values and effect size measures and were guided by the latter. Cramer’s V measures the effect size and the values were interpreted according to Rea and Parker [36], namely Cramer’s V values less than 0.1 are interpreted as insignificant, between 0.1 and 0.2 as weak, between 0.2 and 0.4 as moderate, between 0.4 and 0.6 as relatively strong and above 0.6 as a strong association.

Geographic regions were defined according to the United Nations Statistics Division definition (UNSD): Eastern Europe: Belarus, Bulgaria, Czech Republic, Hungary, Poland, Romania, Russian Federation, Slovakia, Ukraine; Northern Europe: Denmark, Finland, Ireland, Sweden, United Kingdom; Southern Europe: Croatia, Greece, Italy, Portugal, Serbia, Slovenia, Spain; Western Europe: Austria, Belgium, France, Germany, Netherlands, Switzerland; Western Asia: Israel, Turkey. Data on Gross national income (GNI) per capita in US dollars were derived from the World Bank [38, 39]. Information about the prevalence of obesity in Europe was derived from the Global Health Observatory data repository of the World Health Organisation (WHO) [40].

## Results

### Baseline characteristics of all respondents

Out of 17,232 invitations 5695 valid responses were received (response rate 33.0%). The characteristics of respondents have been described in detail elsewhere [21, 22] and are summarised in Table 1. Notably, the mean age was 49.0 ± 12.0 years; 65.0% (3700/5695) were females; the majority were endocrinologists (90.1%, 5132/5695); 83.1% (4732/5695) treated more than 50 hypothyroid patients per year and 78.8% (4487/5693) had more than 10 years in medical practice.

**Table 1:** Characteristics of respondents who stated that they would use thyroid hormones (TH) as obesity treatment in euthyroid patients resistant to lifestyle interventions

*Respondent characteristics*	*Total; N* = *5,695*	*TH prescribers N* = *290*	*TH non-prescribers N* = *5405*	*p value*
Sex				0.41
Female	3700	182 (4.9)	3518 (95.1)	
Male	1995	108 (5.4)	1887 (94.6)	
Age (yrs)				0.42
≤ 30	282	15 (5.3)	267 (94.7)	
31–40	1375	55 (4.0)	1320 (96.0)	
41–50	1565	89 (5.7)	1476 (92.3)	
51–60	1479	76 (5.1)	1403 (94.9)	
61–70	792	44 (5.6)	749 (94.4)	
≥ 70	202	11 (5.4)	191 (94.6)	
Specialty				< 0.01
Endocrinologists	5132	241 (4.7)	4894 (95.3)	
Non-endocrinologists	563	49 (8.7)	514 (91,2)	
Volume in hypothyroidism treatment (patients/year)				< 0.01
Rarely	158	15 (9.5)	143 (90.5)	
10–50 patients	787	40 (5.1)	747 (94.1)	
51–100 patients	1206	43 (3.6)	1163 (96.4)	
≥ 100 patients	3526	192 (5.4)	3334 (94.6)	
Missing data	18			
Years in professional practice				0.06
≤ 10	1206	45 (3.7)	1161 (96.3)	
11–20	1601	87 (5.4)	1514 (94.5)	
21–30	1476	84 (5.7)	1392 (94.3)	
31–40	1010	47 (4.7)	963 (95.3)	
≥ 40	400	27 (6.8)	373 (92.2)	
Missing data	2			
Private practice				< 0.01
No	4,097	183 (4.5)	3914 (95.5)	
Yes	1598	107 (6.7)	1491 (93.3)	
Academic practice				0.13
No	3514	191 (5.4)	3323 (94.6)	
Yes	2181	99 (4.5)	2082 (95.5)	

Data are the number of patients (percentage) in each group or subgroup

### Baseline characteristics of respondents considering TH for obese patients

Of all 5695 respondents, 290 (5.1%) stated that use of TH may be indicated as a treatment for obesity resistant to life-style intervention (Table 1).

Eighty-three percent (241/290) of those who thought obesity was a potential indication for TH were endocrinologists, while the rest were divided between internal medicine, paediatrics, nuclear medicine, surgery, gynaecology, general practice and others. Nevertheless, the view that TH may be indicated for obesity was more common among non-endocrinologists than endocrinologists (8.7% vs. 4.7%, p < 0.01, Cramer’s V 0.054, 95% CI: 0.032–0.081).

On average, these 290 specialists had been practicing medicine for 23.4 (± 11.8) years. Thirty-four percent (99/290) practiced in academic centres, while more than a third (36.9%, 107/290) stated that they also practiced privately.

### Demographics

Age and sex of respondents as well as years in professional practice were not associated with the use of TH in euthyroid obese patients. However, volume of thyroid disease management did impact: thyroid specialists who treated more hypothyroid patients per year were less likely to consider TH for obese patients than those who treated fewer patients with hypothyroidism (p < 0.01). Respondents working in private practice considered obesity as a potential indication for TH more frequently than those in the public sector (6.7% vs. 4.5%; p < 0.01, Cramer’s V 0.046, 95% CI: 0.024–0.073), while no association was found with working in an academic or non-academic environment (4.5% vs. 5.4%; p = 0.13). Being member of an international Thyroid Society increases the chance of using TH to treat obesity, but Cramer’s was very low (data not shown) (Table 1).

### National and regional variations

**Fig. 1:**
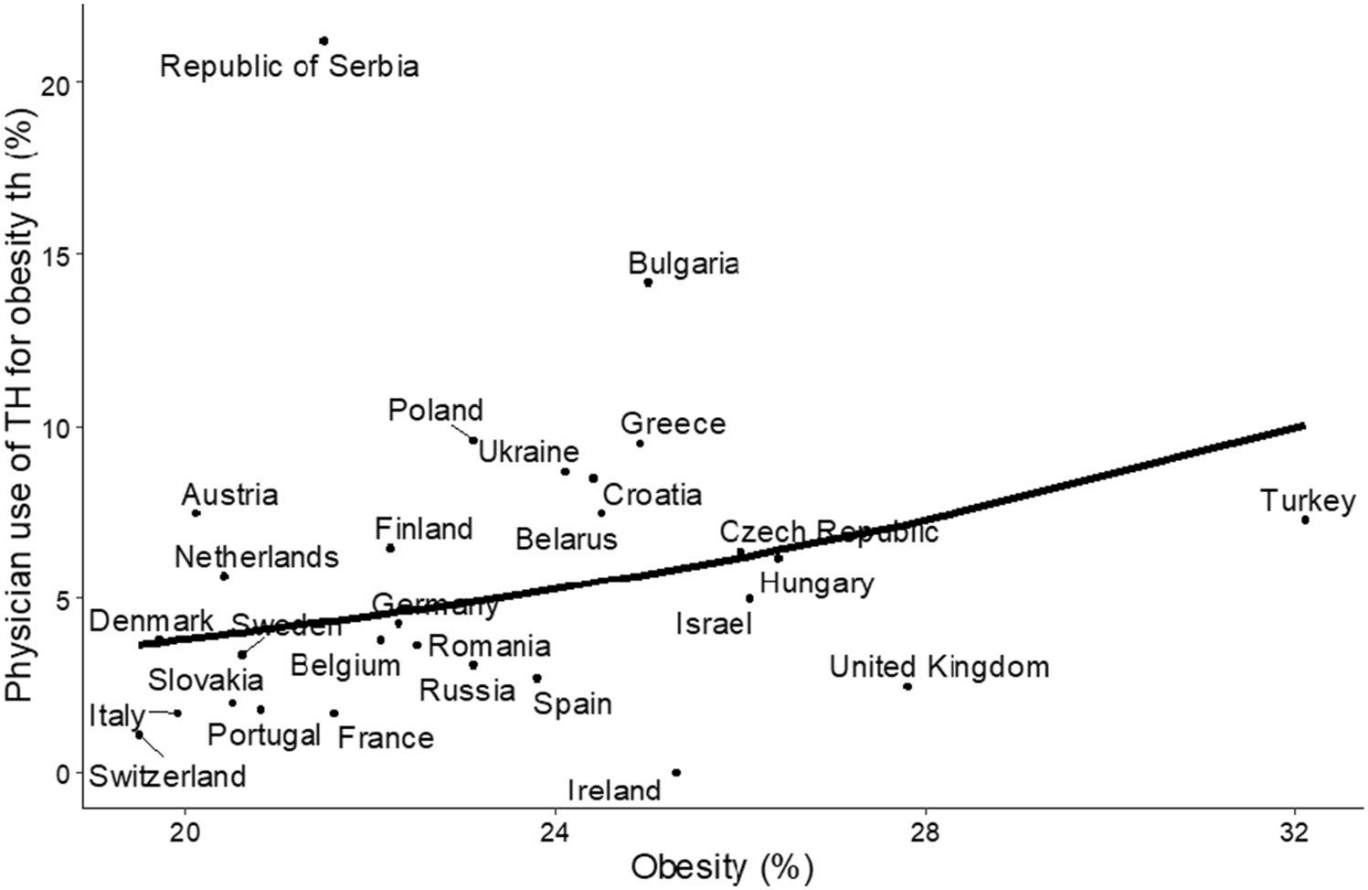
Physicians’ use of thyroid hormone (TH) for obesity and prevalence of obesity in countries included in the study. The line represents predicted probabilities obtained by univariable logistic regression (OR 1.09, 95%, CI 1.05–1.13 per 1 kg/m^2^, p < 0.001). Please note that the line is slightly curved. Using a linear regression plot is not suitable as we analysed the binomial (Yes–No) variable and the probability of obtaining “Yes” for different levels of obesity

There were marked national and regional variations in respondents’ views on obesity as an indication for TH use. The highest attitude to this use were from Serbia (21.2%) and Bulgaria (14.2%), and the lowest from Portugal (1.8%), France and Italy (both 1.7%), Switzerland (1.1%) and Ireland (0%) (Table 2). There were significant differences between Eastern and Western European respondents (7.3% vs. 2.7%, respectively, p < 0.01). The other three regions showed intermediate responses. In general, respondents from Northern and Western Europe were less likely to regard obesity as an indication for TH use than those from Eastern Europe (p < 0.01, Cramer’s V 0.076 95% CI: 0.053–0.103) (Fig. 1).

**Table 2:** Percentage of respondents who use thyroid hormones for obesity in European regions and countries

Country	Responders (N)	N (%)
Eastern Europe	1679	122 (7.3)
Belarus	146	11 (7.5)
Bulgaria	120	17 (14.2)
Czech Republic	157	10 (6.4)
Hungary	160	10 (6.2)
Poland	425	41 (9.6)
Romania	296	11 (3.7)
Russian Federation	131	4 (3.1)
Slovak Republic	49	1 (2.0)
Ukraine	195	17 (8.7)
Northern Europe	713	25 (2.5)
Denmark	158	6 (3.8)
Finland	123	8 (6.5)
Sweden	116	4 (3.4)
Ireland	39	0 (0.0)
United Kingdom	277	7 (2.5)
Southern Europe	2053	98 (4,8)
Croatia	71	6 (8.5)
Greece	441	42 (9.5)
Italy	843	14 (1.7)
Portugal	109	2 (1.8)
Serbia	99	21 (21.2)
Spain	490	13 (2.7)
Western Europe	938	25 (2,7)
Austria	40	3 (7.5)
Belgium	79	3 (3.8)
France	528	9 (1.7)
Germany	161	7 (4.3)
Netherlands	35	2 (5.7)
Switzerland	95	1 (1.1)
Western Asia	312	20 (6.4)
Israel	119	6 (5.0)
Turkey	193	14 (7.3)

### Prevalence of obesity

**Fig. 2:**
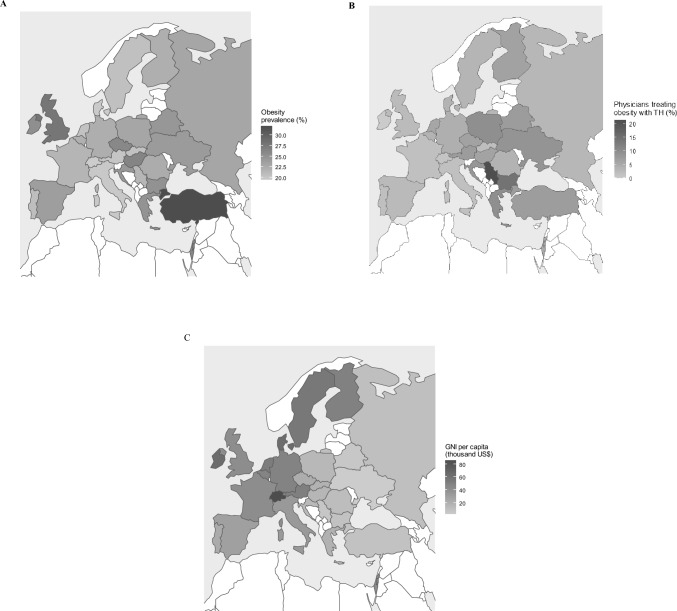
Geographical inequalities in the **A** Prevalence of obesity in Europe by country. Source WHO [Ref 41]. **B** Percentage of specialists who manage obesity with thyroid hormones in Europe according to the THESIS survey (data in Table 2). **C** Gross national income per capita in the countries included in the THESIS survey. Source the World Bank [Ref 40]

The view that TH treatment may be indicated in obese euthyroid patients was positively associated with published data on the prevalence of obesity in different countries (OR 1.09, CI: 1.05 to 1.13; p < 0.001) [41] (Figs. 1 and 2).

### Gross national income and use of TH in obesity

**Fig. 3:**
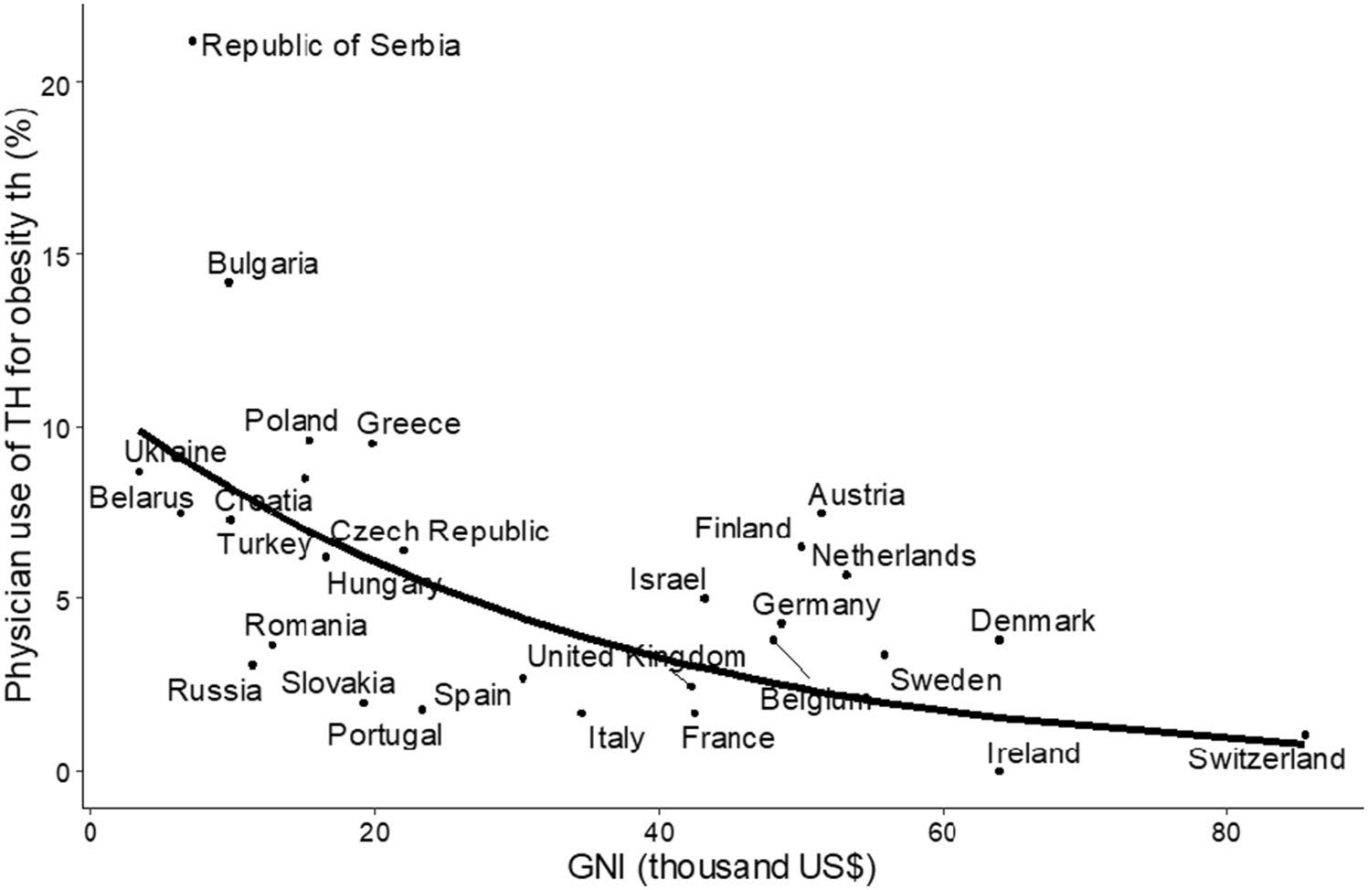
Physicians’ use of thyroid hormone (TH) for obesity and Gross national income (GNI) in countries included in the study. The line represents predicted probabilities obtained by univariable logistic regression (OR 0.97, 95% CI 0.96–0.98 per 1000 US $, p < 0.001). Please note that the line is curved. Using a linear regression plot is not suitable as we analysed the binomial (Yes–No) variable and the probability of obtaining “Yes” for different levels of GNI

**Fig. 4:**
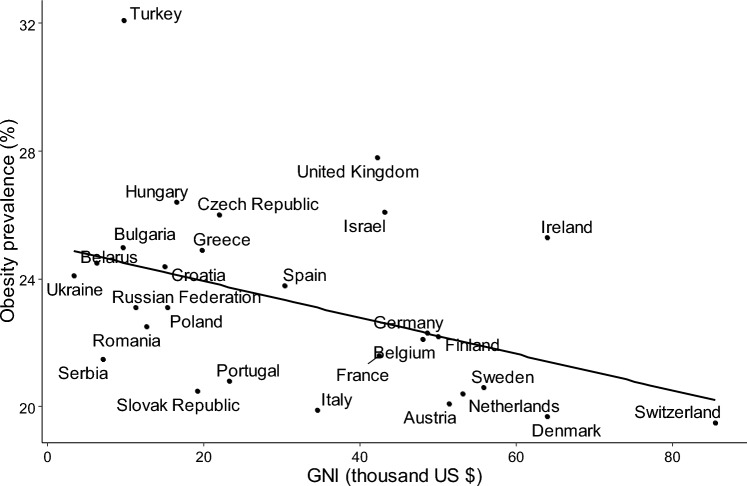
Obesity prevalence and Gross national income (GNI) in countries included in the study. It’s worth noting that in this case, a linear regression is appropriate as we correlated Obesity and GNI per country. The choice of regression method was based on the nature of the variables and the research question we were trying to answer

The propensity to express the view that TH may be indicated in obesity correlated with decreasing GNI (OR 0.969, CI: 0.961–0.977; p < 0.001, per 1000 US$). Interestingly, regardless of the medical practices found in the present investigation, we observed that the prevalence of obesity was inversely correlated with the GNI (Figs. 3 and 4).

### National guidelines and use of TH in obesity

Of the 28 participating countries, 22 provided information on the existence of national guidelines for thyroid disease and obesity. Additional information relating to four countries was obtained from websites of national professional organisations. Information about Ukraine and Belarus could not be obtained. Countries lacking national guidelines for thyroid disease mostly followed the two major international guidelines (European Thyroid Association and American Thyroid Association).

Twenty of the 28 countries represented in THESIS had national guidelines for obesity, and 13 for hypothyroidism. None of the published guidelines (either national or international) on thyroid disease and obesity recommend treating obesity with TH (Table S1, supplementary material). The availability of nationally endorsed obesity guidelines did not influence views on the use of TH for obesity in either univariate or multivariate models. However, the presence of thyroid guidelines was associated with unfavourable views on use of TH for obesity (univariate OR 0.68, 95%, CI 0.53–0.88, p = 0.002; multivariate OR 0.71, 95%, CI 0.55–0.91, p = 0.008). (Tables S1 and S2).

## Discussion

In this study we found that around 5% of European thyroid specialists still consider obesity as a potential indication for TH use. This figure is small and reflects that most specialists follow the indications of TH treatment based on evidence, as no national or international guidelines on obesity or hypothyroidism endorse the use of TH for euthyroid obese patients (Table S1). The universal recommendation against the use of TH in obesity in this scenario is based on the absence of evidence of favourable outcomes and the potential for causing more harm than benefit [42]. Therefore, it is alarming that use of TH for obesity reached up to 14 to 21% in some European countries, such as Bulgaria and Serbia. Given the high prevalence of obesity in Europe, the inappropriate use of TH could potentially harm significant number of euthyroid obese patients. Previous studies have shown that even in hypothyroid patients, there is a high risk of overtreatment leading to increase cardiovascular disease and mortality [43], so one could speculate that treating euthyroid patients with TH is likely to lead to overtreatment.

Our findings are novel in that the tendency to treat euthyroid obese patients with TH was associated with respondent characteristics (non-endocrinologists, working in private practice, treating a small number of hypothyroid patients per annum, practicing in a non-academic environment), and broader national and regional characteristics (prevalence of obesity, Eastern Europe, low GNI, absence of endorsement of thyroid guidelines by national professional societies).

It is interesting to note that specialist views on the use of TH treatment for obesity correlated positively with the national prevalence of obesity. In addition, although speculative, it is possible that an easier access to expensive anti-obesity therapies could impact on the differences noted between treatments in countries with different GNI [44]. In our analysis, GNI correlated inversely with views favouring TH treatment in obese patients. It is well established, that obesity is more prevalent in low income populations, especially in Western countries [45]. This is probably due to easy access to lower-priced, high-calorie food in these societies. At a national level, the prevalence of obesity decreases as the GNI rises [44]. Information on the relationship between hypothyroidism and GNI is scarce. A recent Spanish cross-sectional study found that low-income or unemployed people have a higher frequency of hypothyroidism and hyperthyroidism than more privileged socioeconomic groups [46]. Potential explanations, although cause-effect relationship is difficult to prove, include the bi-directional association between thyroid dysfunction and excess morbidity, whether somatic [47] or psychiatric [48], leading to reduced physical activity. But also the higher unemployment rate [49] and the lower income and thereby impaired access to healthier and more expensive food might play a role.

We found significant geographic differences. Respondents from Northern and Western European countries were clearly less prone to consider TH in obesity than respondents from other countries. In Southern European countries, however, the percentage of respondents who considered TH treatment in obesity rose from 2.5–2.7% to 4.8% compared to Northern and Western Europe. Within Southern Europe, two distinct groups of countries can be identified. Percentages of proponents of TH for obesity in Italy, Portugal, and Spain were very similar to those found in Northern and Western Europe, while values were much higher in Croatia, Greece and Serbia (8.5, 9.5 and 21.2% respectively). Multivariate analysis showed that using TH to treat obesity is associated with geographic region, practicing in private clinic and obesity prevalence in the area. There was no relation between male to female respondents’ ratio and use of TH to treat obesity.

### Plausible explanations for the use of TH in obesity

While it is correct to assume that hypothyroidism is associated with weight gain, decreased thermogenesis and metabolic rate [50], it does not directly follow that the reverse is true. Obesity is usually not associated with hypothyroidism [51]. However, blood TSH levels increase in parallel with increasing BMI in the euthyroid population, and levothyroxine requirement increases in obese hypothyroid patients [25, 27]. The mechanism behind this increase in TSH in obese populations is poorly understood, but it seems to be a consequence rather than the cause of obesity and reverses with restoration of normal BMI through lifestyle changes [52]. It has been speculated that it probably represents a process of adaptation to nutritional status or an alteration of thyroid structure; actually a differential gene expression profiling of metabolic and immune pathways in thyroid tissues of patients with obesity has been observed [53]. However, the relationship between serum TH and obesity is not a constant finding: an observational study in a cohort of more than 400 euthyroid individuals could not confirm this association between serum TH levels and obesity [54].

Despite the absence of a rational scientific basis, TH have been used in the past in attempts to induce weight loss in obese euthyroid subjects [27]. This practice has two major drawbacks. TH supplementation generally does not result in weight loss [55] and can induce iatrogenic thyrotoxicosis in these individuals, which is associated with adverse health outcomes such as increased risk of fracture [56] and excess cardiovascular risk [43]. Therefore, TH therapy should be discouraged in euthyroid patients [57].

The characteristics associated with those respondents recommending TH for euthyroid obese individuals is novel and intriguing. Shortfalls in fulfilment of educational needs, inexperience, professional antagonism, low salaries for medical practitioners and lack of guidance by professional organisations in some countries are some of the physician-related factors that may be relevant. Undoubtedly, patient expectations and pressure on physicians may also play a role.

### Relevance, strength and limitations of the THESIS study

The THESIS collaboration is the largest survey conducted on the use of TH, as for number of respondents as well as participating countries. The aggregate responses are likely to be representative of European practices in secondary care [21, 22]. The response rate was comparable to other studies that have used similar online survey systems [58]. Thus, the THESIS data offer an insight about current use of TH by European specialists. The same study is currently being extended to other continents outside Europe. In fact, we already have published data on the use of TH in Latin America [59] and Australia [60].

Limitations include selection bias inherent in volunteering to participate in surveys. Factors that were not accounted for and which may have influenced the responses include availability of other treatments and services for obesity, previous failure of anti-obesity treatments apart from lifestyle intervention, and patients’ pressure and demand for treatment. In addition, the volume of patients with obesity that seek specialist consultation was not explored. Finally, THESIS did not include primary care physicians, which are usually the first to treat hypothyroidism and see obese patients [61]. However, the real-life practices of the thyroid expert respondents, many of whom are national opinion leaders, are likely to be mirrored in primary care. Therefore, our results contain comprehensive information on the use of TH in obesity in Europe.

## Conclusions

Most thyroid specialists (95%) follow evidence and guidelines in the use of TH for obesity. However, about 5% stated that TH use may be indicated as a treatment for obesity in euthyroid patients resistant to lifestyle interventions. This opinion was associated with (i) respondent characteristics (being non-endocrinologist, working in private practice, treating a small number of hypothyroid patients per year) and (ii) national characteristics (prevalence of obesity, practicing in Eastern Europe, low GNI and absence of endorsement of thyroid guidelines by national professional societies). Our findings raise questions about the underlying drivers, and highlight concerns about ethical and safe use of TH by some thyroid specialists in Europe.

## Supplementary Information

Below is the link to the electronic supplementary material.

Supplementary file1 (DOCX 33 KB)

Supplementary file2 (DOCX 29 KB)

## Declarations

### Informed concent

For this type of study, formal consent is not required.
