# Supplementary material for: Treatment of Obesity with Thyroid hormones in Europe. Data from the THESIS* Collaboration
Source: J Endocrinol Invest. 2024 Jun 15;48(1):201–12. doi: 10.1007/s40618-024-02409-z (PMC11729071; doi:10.1007/s40618-024-02409-z)
Supplement: Supplementary file 1 — Supplementary file1 (DOCX 33 KB) [file 40618_2024_2409_MOESM1_ESM.docx]

Table S1: (supplementary material):

National Guideline (NG) Status by Countries

| *Country* | | *NG Thyroid* | *NG Obesity* | *International^#^* | *Endorse thyroid hormones in obesity* | *Comments** |
| --- | --- | --- | --- | --- | --- | --- |
| Eastern Europe | |  |  |  |  |  |
|  | Belarus |  |  |  |  | NA |
|  | Bulgaria | Yes | Yes |  | Not mentioned |  |
|  | Czech Republic | Yes | Yes |  | Against |  |
|  | Hungary | Yes | Yes |  | Not mentioned |  |
|  | Poland | No | Yes | Yes (ESE, ETA, ATA) | Not mentioned |  |
|  | Romania | No | No |  | Not mentioned |  |
|  | Russian Federation | Yes | Yes |  |  | Webpage |
|  | Slovak Republic | Yes | Yes |  |  |  |
|  | Ukraine |  |  |  |  | NA |
| Northern Europe | |  |  |  |  |  |
|  | Denmark | Yes | Yes |  | Not mentioned |  |
|  | Finland | No | No | Yes (ETA, ATA) | Not mentioned |  |
|  | Sweden | No | Yes | Yes (ETA, ATA) | Not mentioned |  |
|  | Ireland | No | Yes |  | Not mentioned |  |
|  | United Kingdom | Yes | Yes |  | Against |  |
| Southern Europe | |  |  |  |  |  |
|  | Croatia | No | Yes |  |  | Webpage |
|  | Greece | No | No | Yes (ETA, ATA) | Not mentioned |  |
|  | Italy | Yes | Yes | Yes (ATA) | Not mentioned |  |
|  | Portugal | Yes | Yes |  | Against |  |
|  | Serbia | Yes | Yes |  | Against |  |
|  | Spain | No | No | Yes (ETA, ATA) | Not mentioned |  |
| Western Europe | |  |  |  |  |  |
|  | Austria | No | No | Yes (international) | Not mentioned |  |
|  | Belgium | No | Yes |  | Against |  |
|  | France | Yes | Yes |  | Not mentioned |  |
|  | Germany | No | Yes | Yes (ETA, ATA) | Against |  |
|  | Netherlands | Yes | Yes |  |  | Webpage |
|  | Switzerland | No | Yes |  |  | Webpage |
| Western Asia | |  |  |  |  |  |
|  | Israel | No | No | Yes (ATA) | Against |  |
|  | Turkey | Yes | Yes |  | Not mentioned |  |

^#^ International: international guidelines followed by countries without national guidelines. ESE: European Society of Endocrinology; ETA: European Thyroid Association; ATA: American Thyroid Association. For the analysis “not mentioned” was interpreted equal as “against”. * The information provided in this column was obtained from the web pages of the national endocrinology organizations because the national leaders failed to provide the required data. NA: information not available due to Internet blackout.
