# Supplementary material for: Treatment of Obesity with Thyroid hormones in Europe. Data from the THESIS* Collaboration
Source: J Endocrinol Invest. 2024 Jun 15;48(1):201–12. doi: 10.1007/s40618-024-02409-z (PMC11729071; doi:10.1007/s40618-024-02409-z)
Supplement: Supplementary file 2 — Supplementary file2 (DOCX 29 KB) [file 40618_2024_2409_MOESM2_ESM.docx]

Table S2: (supplementary material):

| Variable | Univariable OR | Multivariable OR |
| --- | --- | --- |
| 11-20 Years of practice* |  | 1.94; 95% CI 1.31-2.88  p <0.01 |
| 21-30 Years of practice* |  | 2.01; 95% CI 1.35-2.99  p <0.01 |
| 31-40 Years of practice* |  | 1.68; 95% CI 1.08-2.62  p=0.022 |
| >40 Years of practice* |  | 2.33; 95% CI 1.38-3.94  P=0.002 |
| Endocrinologist |  | 0.49; 95% CI 0.35-0.69  p <0.01 |
| GNI thousand US$ |  | 0.97; 95% CI 0.96-0.98  p <0.01 |
| Thyroid national guidelines exists | 0.68; 95% CI 0.53-0.87  p 0.002 | 0.71; 95% CI 0.55-0.91  p=0.008 |

* Compared to ≤ 10 Years of practice

Univariable and multivariable logistic regression for using TH to treat obesity in euthyroid subjects. Multivariable analysis includes all univariable analysis.
